# Titanium Dioxide Microscale and Macroscale Structures: A Mini-Review

**DOI:** 10.3390/nano10061190

**Published:** 2020-06-18

**Authors:** Vu Khac Hoang Bui, Vinh Van Tran, Ju-Young Moon, Duckshin Park, Young-Chul Lee

**Affiliations:** 1 Department of BioNano Technology, Gachon University, 1342 Seongnamdaero, Sujeong-gu, Seongnam-si, Gyeonggi-do 13120, Korea; hoangvu210190@gmail.com (V.K.H.B.); vanvinhkhmtk30@gmail.com (V.V.T.); 2 Department of Beauty Design Management, Hansung University, 116 Samseongyoro-16gil, Seoul 02876, Korea; bora7033@naver.com; 3 Korea Railroad Research Institute (KRRI), 176 Cheoldobakmulkwan-ro, Uiwang-si, Gyeonggi-do 16105, Korea

**Keywords:** TiO_2_, macroscale structures, microscale structures, environmental treatment, photocatalytic

## Abstract

Titanium dioxide nanoparticles (TiO_2_ NPs) have some limitations, such as their low surface area, high bandgap energy, and low recycling ability. To overcome these limitations, TiO_2_ can be prepared in microscale/macroscale structures. TiO_2_ microscale structures, in comparison with TiO_2_ nanopowder, have higher surface areas, more tunable pore structures, and better top photocatalytic activity. In contrast, for TiO_2_ macroscale structures, although the surface area is lower than TiO_2_ nanopowder in many cases, they still achieve similar or better photocatalytic performance due to their unique properties. Moreover, both TiO_2_ microscale and macroscale structures can be easily recovered from reaction media. The difference between these two types of TiO_2_ structures is a function not only of size but also of the preparation process. Every type of TiO_2_ structure has its own advantages and disadvantages, as will be discussed further in the following pages. Future perspectives on this research field also will be discussed.

## 1. Introduction

Titanium dioxide nanoparticles (TiO_2_ NPs) are widely applied in various areas, such as wastewater treatment, dye-sensitized solar cells (DSSCs), lithium-ion batteries (electrodes), chemical sensing, hydrogen production, antimicrobial applications, and cosmetics [1,2,3,4]. TiO_2_ is an n-type semiconductor due to its oxygen deficiency [5]. It has three types of polymorphs, including tetrahedral anatase, rutile, and orthorhombic brookite. Among them, anatase TiO_2_ NPs have the highest photocatalytic activity due to the retardancy of the recombination of holes and electrons [6,7]. The energy bandgaps of anatase, rutile, and brookite are 3.2, 3.0, and ~3.2 eV, respectively [8,9,10]. Many studies have claimed that the combination of the anatase and rutile phases at a suitable ratio has higher photocatalytic activity than the single anatase or rutile phase [11,12,13,14,15]. TiO_2_ NPs can be produced via different methods, such as sol–gel, hydrothermal or solvothermal, pulsed laser deposition, chemical decomposition (CVD), chemical vapor decomposition, micelle and inverse micelle, direct oxidation, or sonochemical methods [16,17,18]. The advantages of TiO_2_ NPs over other photocatalytic semiconductors come from their photostability, low-cost of production, as well as chemical and biological inertness [19,20].

There are three basic steps in photocatalysis: light absorption, charge separation, and surface reaction [21]. When photons are irradiated by sunlight, which has an energy equal to or exceeding the optical bandgap (E_g_) of a photocatalyst, the excited electron moves from the valence band to the conduction band, leaving a hole in the valence band as it does so. This phenomenon is called “charge separation”. Photogenerated electrons and holes can either recombine or react with electron donors or acceptors to produce different reactive oxygen species (ROS), such as •O_2_^−^, •OH, and •OOH, which have the capacity to remove pollutants from water and air [7,22]. Among these ROS, •OH is the most powerful oxidizing species, second only to fluorine [23,24].

The photocatalytic mechanism is represented by the following chemical equations [8]:

TiO_2_ + h*v* → h_vb_^+^ + e_cb_^−^

H_2_O + h_vb_^+^ → ●OH + H^+^

O_2_ + e_cb_^−^ → ●O_2_^−^

●OH + pollutant → H_2_O + CO_2_

●O_2_^−^ + H^+^ → ●OOH

●OOH + ●OOH → H_2_O_2_ + O_2_

●O_2_^−^ + pollutant → H_2_O + CO_2_

●OOH + pollutant → H_2_O + CO_2_.

**Figure 1:**
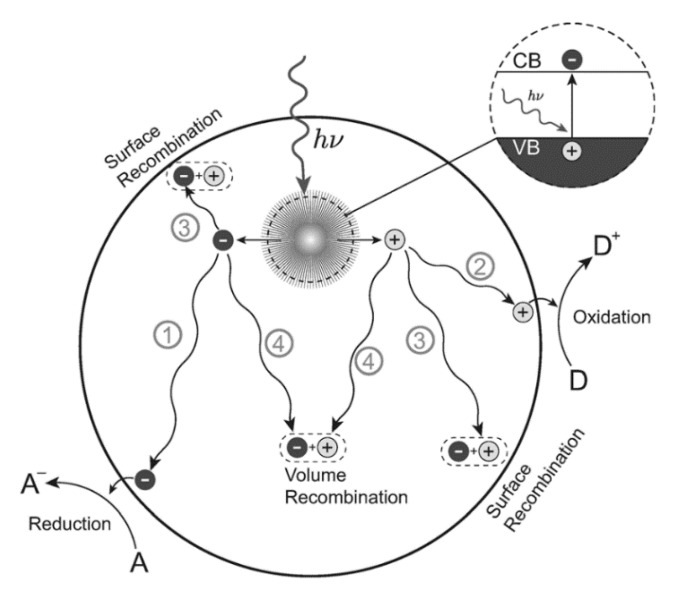
Formation of electron–hole pairs in semiconductor materials. Reprinted with permission from [33]. Copyright 2013, Wiley.

In photocatalytic reactions, the reactants are diffused and absorbed at actives sites of TiO_2_ NPs. Then, the products are formed on the surfaces of TiO_2_ NPs via the arrangement of the reactant surfaces and charge exchange between TiO_2_ NPs and pollutants. These products are consequently desorbed and diffused to the surrounding environment [21]. This photocatalytic mechanism is schematized in Figure 1. In addition, oxygen vacancies thus formed can function as photo-excited electron–hole pair separators, thereby enhancing photocatalytic activity [25,26]. Additionally, the formation of oxygen vacancies could lead to the creation of unpaired electrons or centers of Ti^3+^, which could form donor levels in the TiO_2_ electronic structure [27]. Defective TiO_2_ with oxygen vacancies could be obtained via different processes, such as hydrogen thermal treatment, high energy particle bombardment, doping of metals or non-metals, or thermal treatment under oxygen-depleted conditions [28]. Moreover, oxygen vacancies can be formed under photocatalytic reaction [29]. However, high-density oxygen vacancies can act as charge recombination centers, thus decreasing the mobility of free carriers and photocatalytic performance [30,31,32].

Besides their advantages, TiO_2_ NPs have three main limitations: fast recombination of electron–hole pairs, poor light-source utilization, and difficulty in recycling [34]. The limitations of the large energy bandgap and the fast recombination of electron–hole pairs can be overcome by different strategies, such as coupling with a narrower bandgap semiconductor, doping or co-doping with metal or non-metal ions, surface sensitization by metal complexes or organic dyes, deposition of noble metals, surface fluorination, and surface sulfation [35]. Another limitation is related to the low recycling utility of TiO_2_ NPs, which could result in secondary pollution problems [36]. TiO_2_ NPs’ recycling limitation can be overcome by their immobilization on different substrates [37,38]. A serious drawback of this strategy, however, is the detachment of TiO_2_ NPs from their carrier substrates via hydraulic blow and collision [39].

Compared with powder TiO_2_ NPs, TiO_2_ microscale structures have a higher surface area and tunable pore structure [40,41]. According to its narrower and uniform pore size distribution, TiO_2_ microscale structures have high surface areas and a uniform porous structure compared with powder TiO_2_ NPs [42]. Due to their unique properties, TiO_2_ microscale structures display higher catalytic activities than powder TiO_2_ NPs in most cases [43,44,45,46,47,48]. Beside their higher surface area and tunable pore structure, another remarkable reason is the ability to multiscatter incident light, which leads to higher light utilization and the suppression of charge separation [21]. TiO_2_ microscale structures are also easy to be recovered and repeatedly utilized [49]. TiO_2_ microscale structures such as hollow spheres offer the potential for utilization not only in environmental treatment contexts, but also in other applications, such as controlled-release capsules, artificial cells, and drug delivery [50].

In contrast, TiO_2_ macroscale structures mostly have a lower surface area than powder TiO_2_ NPs [32,51]. The sintering processes at a high temperature can explain the reduction of the surface area of TiO_2_ pellets [32,52,53]. However, compared to TiO_2_ NPs, TiO_2_ macroscale structures such as TiO_2_ porous ceramic pellets have more optical activity centers and more carriers, while the photocatalytic activity lasts for a longer time [51]. In techniques such as hot isostatic pressing (HIPing), the contamination of carbon could lower the bandgap and increase the photocatalytic performance [1]. In addition, TiO_2_ macroscale structures are easy to recover from the media due to big size. In the case of TiO_2_ floating structures, the photocatalytic activity is higher than the powder form [54]. One remarkable reason is their ease in directing exposure to light sources [34,54].

## 2. TiO_2_ Microscale Structures

### 2.1. TiO_2_ Solid Microscale Structures

TiO_2_ solid spheres can be synthesized via various methods, such as through addition of surfactants, hydrothermally, spray-drying, freeze-drying, templating, or just by modifying the order of reactant addition.

Different ionic and non-ionic surfactants have been used to synthesize mesoporous materials [55]. Wang et al. (2000) synthesized mesoporous TiO_2_ spheres via the slow hydrolysis of titanium alkoxide with neutral surfactant dodecylamine as a template under the condition of environmental humidity. They suggested that the surfactant takes on more important roles in the formation of the mesoporous structure than in the formation of spherical morphologies. In contrast, under low concentrations of titanium tetraisopropoxide Ti(OPr)_4_, the spherical shapes of TiO_2_ are more favorable in the reaction system. The template was removed by diluting 0.3 g of sample in a mixture of ethanol (40 mL) and HCl (1 mL). The presence of an acidified ethanol extraction process is necessary. The obtained TiO_2_ mesoporous spheres had a spherical form and smooth surfaces. The sizes of obtained materials ranged from hundreds of nanometers up to several micrometers, with Brunauer–Emmett–Teller (BET) surface areas of 115 m^2^/g, specific pore volume of 0.19 cm^3^/g, and an average pore diameter of 5.4 nm. They found that static treatment is critical and that the synthesis of TiO_2_ materials by stirring or dropping water results in particles lacking specific shape [56].

**Figure 2:**
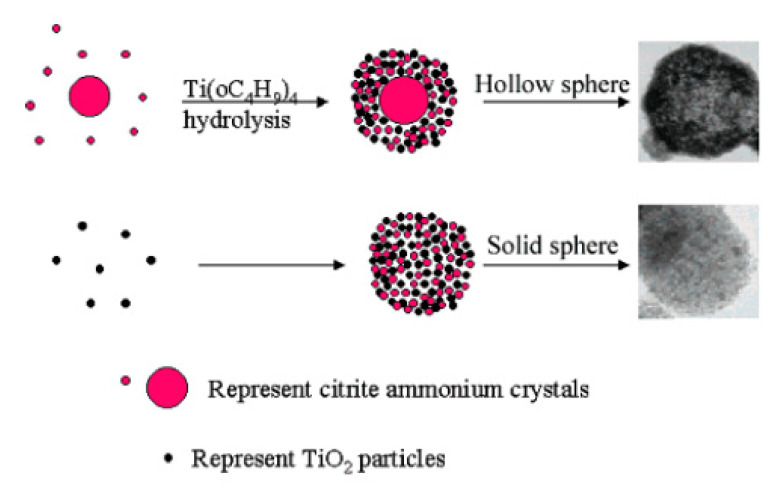
Scheme of mesoporous hollow and solid spheres formation. Reprinted with permission from [50]. Copyright 2005, American Chemistry Society.

However, TiO_2_ spheres can also be obtained without the use of surfactants or templates. Zhang et al. (2005) prepared both solid TiO_2_ spheres (200–300 nm) and hollow TiO_2_ spheres (200–500 nm) simply by changing the order of reactant addition. In their preparation of TiO_2_ solid spheres, titanium butoxide (TB, 6 mL) was dissolved in absolute ethanol (40 mL). Then, citric acid (0.0015 mol), distilled (DI) water (2 mL), and NH_3_●H_2_O (20 mL) were successively added to the above solution. The solution was stirred for several hours and left to stand overnight. Subsequently, the white precipitate was filtered, washed with DI water and ethanol, and dried at 60 °C for 8 h. Lastly, the powder was sintered at 500 °C for 4 h. As for the mesoporous TiO_2_ hollow spheres, citric acid (0.0015 mol) was first dissolved in ethanol solution (40 mL) and DI water (2 mL). Afterward, NH_3_●H_2_O (10 mL) was added to the mixture solution to form and grow ammonium citrate crystals. Lastly, TB (6 mL) and ammonium (10 mL) were added to the mixture solution at the same time. The dipping rate for ammonia is about two times that of TB. The following steps were the same for both solid and hollow TiO_2_ spheres. After the calcination process, the spheres were composed of small particles (7 nm) and formed mesoporous structures (a disordered wormhole framework) that could not be seen before calcination. Zhang et al. (2005) explained that ammonium citrate plays an important role in mesoporous sphere formation, in that mesoporous TiO_2_ solid or hollow sphere formation is highly influenced by the extent of TiO_2_ condensation that exists at the beginning of ammonium citrate crystal growth. Therefore, they fabricated mesoporous solid spheres simultaneously with the TiO_2_ condensation process and the formation of ammonium citrate crystals. In contrast, mesoporous hollow spheres were formed in the presence of ammonium citrate crystal growth and the TiO_2_ condensation process, in order (Figure 2). Additionally, both the TiO_2_ solid and hollow spheres had a mesoporous structure with average pore sizes of 6.8 and 7.0 nm; and average BET surface areas of 162 and 90 m^2^/g, respectively. The bandgap energy values of the TiO_2_ solid and hollow spheres were 3.68 and 3.75 eV, respectively [50].

The pulsed laser ablation in liquid (PLAL) technique is easy, fast, and eco-friendly. Balati et al. (2019) recently applied the PLAL technique to prepare black titanium dioxide with a TiO_2_ rutile microsphere as the core and hydrogenated anatase TiO_2_ as the outer layer. The pristine anatase TiO_2_ was added into DI water and irradiated with the laser ablation for 5–120 min. The maximum particle size growth was obtained when the sample was irradiated for 120 min. The photocatalytic reaction showed that 99% of methylene blue (MB) was removed after 60 min under visible light irradiation. The enhancement of visible light absorption and the increase of charge carrier lifetime according to the formation of different types of heterojunctions could be explained by the high photocatalytic performances. In addition, the hydroxyl radical (●OH) was proven to act as the main active species in the photocatalytic reaction [57].

TiO_2_ NPs can also be packed into granule form by using the spray-drying method. In spray-drying, a hot gas is used to rapidly dry a NP suspension. Afterward, a spray nozzle is applied to distribute the slurry into a controlled drop-size spray. Vicent et al. (2011) used spray-drying techniques to prepare TiO_2_ granules from a P25 nanopowder suspension. The nanosuspension was stabilized by a polyacrylic acid (PAA)-based polyelectrolyte and an ultrasound probe (5 min) was used to increase the solid loading up to 30 vol.%. The obtained granules were spherical and of ~60 µm size and 1335 kg/m^3^ density [58]. Faure et al. (2010) used the spray-drying technique to prepare redispersible granules with a size between 20 and 50 μm from TiO_2_ NPs. Interestingly, the granules could be converted to TiO_2_ NPs with a size distribution similar to TiO_2_ powder by ultrasonication [59]. Pal et al. (2014) also used a spray-drying method to prepare TiO_2_ microspheres with a diameter of 2 to 10 μm from a hydrothermally cured aqueous suspension of TiO_2_ nanoparticles. The obtained TiO_2_ microspheres had both anatase and rutile phases. It was shown that the rutile fraction increases with annealing temperature and dominates anatase when the annealing temperature was over 500 °C. Compare with TiO_2_ powder, TiO_2_ microspheres showed higher photocatalytic activity towards rhodamine B (RhB), MB, and methyl orange (MO). TiO_2_ microspheres obtained with an annealing temperature of 400 °C showed the highest degradation efficiency [60].

Vicent et al. (2012) compared TiO_2_ granules prepared by freeze-drying and spray-drying. In their study, various parameters such as temperature, pressure, nozzle diameter, and solid loading were evaluated for both methods. They found that only the solids contents of the suspension influenced the morphology and characteristics of dried granules. There were some differences between the TiO_2_ granules prepared from freeze-drying and spray-drying. The TiO_2_ granules from spray-drying had a monomodal distribution with a higher granule size, while those from freeze-drying were more porous, with a bimodal intragranular distribution. Thus, the TiO_2_ granules obtained from spray-drying displayed better flowability (in terms of the Hausner ratio), while those from freeze-drying were softer and of higher porosity [61].

Another popular means of TiO_2_ sphere preparation is the hydrothermal or solvothermal method. Du et al. (2011) prepared TiO_2_ microspheres using the hydrothermal method under different temperatures (140, 160, 180, and 200 °C) and times (0.5, 2, 24, and 36 h). The temperature condition affected the morphology of the obtained TiO_2_ microspheres. At 140 °C, the microspheres were formed with a diameter of about 1–2 µm. When the temperature was increased to 160 °C, the inhomogeneous microspheres were obtained with the largest diameter (~3 µm). At 180 °C, well-defined porous microspheres were obtained and only small ratios of irregular particles could be observed. However, with continued increase of temperature, more irregular particles were seen. The BET surface area of the TiO_2_ prepared at 180 °C was five times larger than that of P25 (265.4 m^2^/g vs. 50 m^2^/g). The optimal reaction time was around 24 h, while the increase of temperature led eventually to the destruction of microsphere structures. The apparent Oswald ripening could be attributed to the formation of the TiO_2_ microspheres. In an air purification application, the optimal TiO_2_ microspheres (temperature: 180 °C, time: 24 h) could covert 90% of benzene to CO_2_ and H_2_O after 50 min. In contrast, the removal efficiency for P25 was only 45% under the sample photocatalytic reaction conditions. In addition, having seen no color change on the surfaces of the TiO_2_ microspheres, the authors concluded that the intermediate products had been completely removed from the environment [42].

Mesoporous TiO_2_ spheres can be synthesized via hydrothermal methods with sodium salicylate as a template, as was accomplished in a previous study [16]. The as-synthesized TiO_2_ was composed of tiny TiO_2_ NPs (12–20 nm). With the entrapping of the photosensitizer inside the mesoporous materials, the obtained TiO_2_ spheres had photocatalytic activity under the irradiation of visible light. Regarding the formation mechanism of TiO_2_ microscale structures via electrostatic interaction, positively charged TiO_2_ NPs could react with negative carboxylate groups of sodium salicylate. The presence of the ortho phenolic-OH group in the salicylate molecule formed a supramolecular assembly among the ligated salicylate moieties under mildly acidic synthesis conditions via hydrogen bonding and hydrophobic interactions. This resulted in the formation of the cage-like structure inside the TiO_2_ nanocrystals. During the calcination process, the template moieties were removed and mesoporous TiO_2_ spheres were formed [16].

Solvothermal synthesis is similar to the hydrothermal methods, but the precursor solution is non-aqueous. Mun et al. (2017) synthesized TiO_2_ spheres by solvothermal methods at different temperatures. They observed that the mixed anatase and rutile spheres were collected at 800 °C. At higher temperatures (≥900 °C), the anatase was transferred to the rutile phase. Such spheres have been applied to produce white-light-emitting diodes (WLEDs) with 43.6% higher light extraction efficiency than WLED combinations of commercial YAG:Ce^3+^ and blue LED chips [62].

Recently, Pulido Melian et al. (2019) used the sol–gel method to synthesize TiO_2_ microspheres. In their study, TiO_2_ microspheres were prepared by hydrolysis and condensation processes from TB precursor and calcinated at 150 °C for 24 h, 400 °C for 1 h, and 630 °C for 1 h. TiO_2_ microspheres calcinated at 150 °C had a diameter of 1.25 μm, while both TiO_2_ microspheres calcinated at 400 °C and 630 °C had a diameter of 1.75 μm. TiO_2_ microspheres were then decorated with Au or Pt particles by photodeposition. They found that TiO_2_ microspheres calcinated at 400 °C and modified with Pt (0.27 wt%) showed the highest production rate of hydrogen (2121 μmol/h) [41].

**Figure 3:**
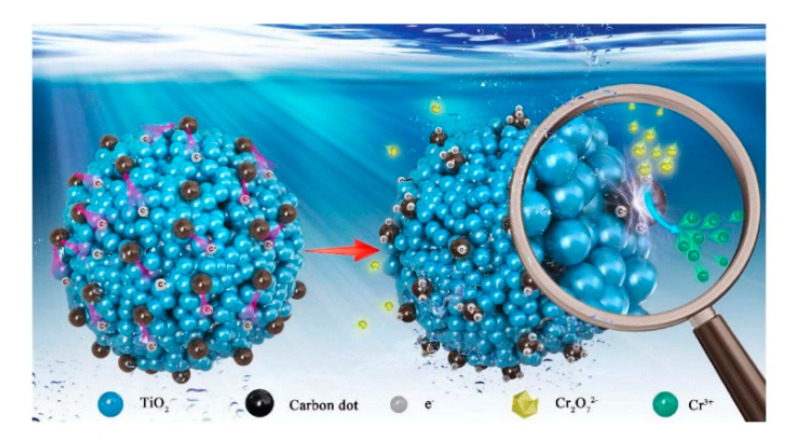
Adsorption–photoreduction–desorption mechanism of Cr(VI) in the presence of the CDs/MT composite. Reprinted with permission from [63]. Copyright 2018, Elsevier.

Besides pure TiO_2_ solid microscale structures, composite TiO_2_ solid microscale structures have been tested. For example, carbon dots (CDs) have been applied to prepare TiO_2_ microscale structures due to their good photoelectric properties [63]. Hydroxyl groups and carboxyl groups are formed on the surfaces of CDs that have high water solubility and suitable chemical reactivity [64,65]. By modification of surface groups, the fluorescent properties of such CDs can be controlled [66,67]. In the study by Zhang et al. (2018), CDs were coupled with TiO_2_ mesocrystals (CDs/MT), where CDs took the role of both electron collectors and active sites (Figure 3). The 0.75 wt% CDs/MT displayed 5.4 times higher activity than the pure TiO_2_ mesocrystals. The loading of CDs did not affect the morphology of the TiO_2_ mesocrystals. The CDs/MT of 0.75 wt% retained 60% of its photocatalytic performance after ten cycles, whereas the pure TiO_2_ mesocrystals retained only 3% of its photocatalytic performance after five cycles. The 0.75 wt% CDs/MT composite had higher durability and stability due to its positive surface, which is an advantage of the removal of Cr(III) cation through the photocatalytic reaction. The reason for the decrease of photocatalytic performance after only a few cycles could be explained by the coverage of the active surface sites by photocatalytic reduction products (Cr(III)). Additionally, with the increase of the amount of CDs, the BET, pore volume (V_p_), and pore diameter of the TiO_2_ microscale structures were slightly reduced. Therefore, the coupling of CDs may cause blockage of pores in TiO_2_ mesocrystals. The positive charges on the CD/MT surface play a role in the selective adsorption of Cr(VI) and rapid desorption of Cr(III), thus improving the photocatalytic reduction of Cr(VI) and the retention of photoreduction activity. The pure TiO_2_ mesocrystals had a Cr(VI)/Cr(III) adsorption capacity ratio of 7.1, while that for the 0.75 wt% CDs/MT composite was 15. Additionally, the existence of CDs on TiO_2_ mesocrystals accelerated the separation of the photogenerated charge. At a pH of 3.0, the 0.75 wt% CDs/MT sample had a zeta-potential of +34.6 mV, higher than the +24.6 mV of the pure TiO_2_ mesocrystals. However, the zeta potential was significantly decreased at the pH of 5.0 to ~ + 8.2 mV for the 0.75 wt% CDs/MT sample, and to +20.5 mV for the pure TiO_2_ mesocrystals samples. Due to this reduction, the 0.75 wt% CDs/MT sample achieved only 65% photoreduction activity as compared to the pure TiO_2_ mesocrystals at the same pH value (5.0) [63].

In addition, TiO_2_ NPs have been coated onto different polymers to form TiO_2_ microscale structures. According to Singh et al. (2013), the advantages of polymer-support TiO_2_ come from the maximal light-utilization efficiency, economic advantages, high degradation efficiency, and easy recovery after photocatalytic reaction [68].

Fabiyi et al. (2000) used a simple thermal treatment method to coat P25 onto expanded polystyrene (PS) for methylene blue (MB) photodegradation. Under thermal treatment (~150 °C), the polystyrene could be expanded 2–4 times larger than its original size, thus lowering its density (from ~0.9 g/cm^3^ to ~0.62 g/cm^3^). These TiO_2_/PS beads could be used to remove MB from an aqueous solution for ten consecutive cycles with a removal efficiency reduced by only about 30%, thus confirming their reuse ability. However, the limitation of this study was the lack of visible light activation of the resulting TiO_2_ microscale structures [37].

In the study by Magalhaes et al. (2009), 18 wt% TiO_2_ was permanently coated onto expanded polystyrene (EPS). A PS solution (10 wt%) in ethyl acetate (EA) was sprayed onto the EPS particles (1 g, 2–4 mm) and TiO_2_ (1 g) was immediately dispersed onto the PS/EA surface. The EA was removed after drying at 80 °C for 1 h and the TiO_2_ particles were immobilized on the EPS surface by a rigid PS layer. This floating TiO_2_/EPS was used for four consecutive cycles without any significant reduction in dye removal efficiency. Interestingly, the total organic carbon (TOC) removal efficiencies even increased after the first cycle. These authors explained that the enhancement of the TOC removal efficiencies could have come from the “aging” process of the catalysts, whereby in the second cycle the catalyst was wetter and had better interaction with the aqueous surface. The TiO_2_ was strongly grafted and could not be removed from the surface of the EPS after 1 h of vigorous stirring in water. However, the surface area of the TiO_2_/EPS (4 m^2^/g) was lower than the P25 powder (45 m^2^/g). Even though TiO_2_/EPS had a lower surface area, it was better than the P25 powder in the photocatalytic test. This could be explained by the precipitation of the P25 powder to the bottom of the reactor, which could not be irradiated by ultraviolet (UV) or solar light. Infrared (IR) spectroscopy analysis also confirmed that the EPS surfaces had not been attacked by the generated ROS during photocatalytic degradation [69].

**Figure 4:**
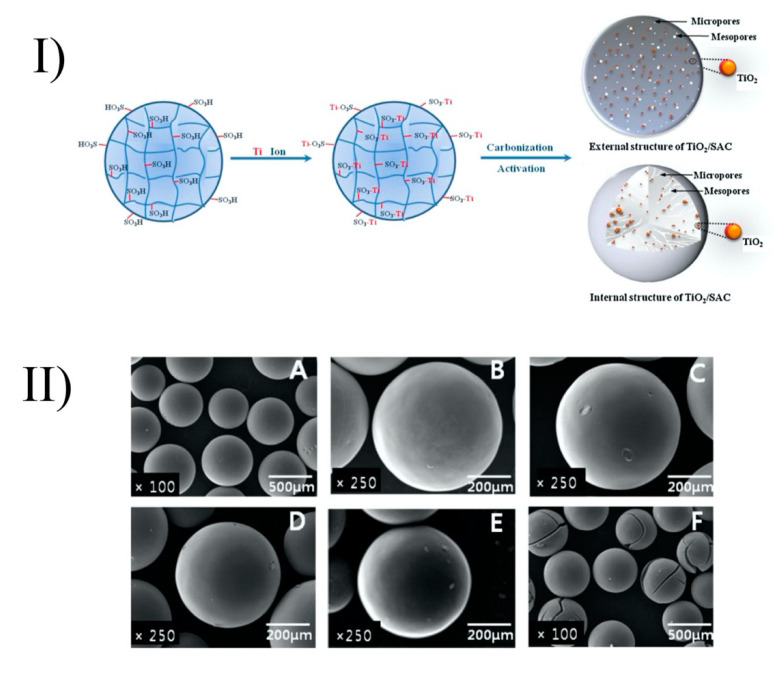
Formation of TiO_2_/SAC spheres (**I**) and their surfaces after heat treatment (**II**) at different temperatures: strong acid ion exchange resin (**A**), TiO_2_/SAC-700 (**B**), TiO_2_/SAC-900–0.5 (**C**), TiO_2_/SAC-900-2 (**D**), TiO_2_/SAC-900-6 (**E**), and TiO_2_/SAC-900-9 (**F**). Reprinted with permission from [70]. Copyright 2013, Elsevier.

Baek et al. (2013) prepared TiO_2_-activated carbon spheres (TiO_2_-SAC) by coating of TiO_2_ onto strong acid ion exchange resin (Diaon SK1BH) (Figure 4). However, with the high activation temperature (900 °C), the peaks of rutile were shown in a powder X-ray diffraction (XRD) analysis. The anatase crystallite size of the TiO_2_-SAC decreased with increasing activation time. Activation time increased the specific surface area and enhanced the porosity. Thus, TiO_2_-SAC with activation times of 6 and 9 h (which are mesoporous spheres) showed adsorption towards humic acid and the best photocatalytic performance. The TiO_2_-SAC with an activation time of 9 h had the same photocatalytic ability as TiO_2_-SAC with an activation time of 6 h, even though it had the highest titanium content (10 wt%) and the largest specific surface area (1427 m^2^/g) and total pore volume (1.2 cm^3^/g). This phenomenon could be explained by the former’s higher proportions of the rutile phase, as mentioned above. From the inductively coupled plasma optical emission spectroscopy (ICP-OES) analysis, the leaching of titanium into the environment after the photocatalytic reaction was negligible. The TiO_2_-SAC spheres exhibited recycling abilities with only a small decrease (~13%) of removal efficiency in the following cycles. By using the exchange method and activation process, TiO_2_ can be immobilized onto ion exchange resin without any binder and can maintain a smooth surface (Figure 4) [70].

Floating structures have some advantages, such as the ability to receive sufficient light energy to produce free radicals [71]. In order to create floating structures, one strategy is the immobilization of TiO_2_ NPs onto different substrates, such as hollow glass beads, exfoliated vermiculite, or EPS beads [69,71,72,73]. In addition, floating photocatalytic composite structures can be prepared by injection of lipid (sunflower oil or liquefied cocoa butter) into the TiO_2_ suspension to control the size of emulsion via the membrane emulsification process. TiO_2_ microscale structures have diameters ranging from 80 to 300 μm. In a previous study, the photocatalytic activity of floating structures was enhanced by the introduction of silver particles. The composite particles based on cocoa butter were shown to be more robust and were not affected by the consequences of the UV photocatalytic reaction. Through the combination of cocoa butter and hexane, the obtained composites floating structures contained 36 mg of TiO_2_ per gram of particle. Interestingly, optimal dye decomposition was achieved with a particle surface coverage of between 60 and 80%. Complete surface coverage affected a reduction in photocatalytic activity due to the reflection of UV light [74].

High-speed granulation can be used to convert powder of nanoparticles into micrometer- or millimeter-sized granules. Goedecke et al. (2017) immobilized TiO_2_ NPs on the surface of SiO_2_ granules using a high shear granulation process with nanozirconia used as the inorganic binder. TiO_2_-coated granules tempered at 300 °C displayed high stability in an aqueous solution up to several hours. The structure with SiO_2_ as the core and TiO_2_ at the outer layer was confirmed by energy-dispersive X-ray spectroscopy (EDX). From SEM images, the thickness of the TiO_2_ layer was around 5–10 μm. Interestingly, the smaller fraction (250–500 μm) with the higher surface area displayed lower photocatalytic activity against MB than the coarse fraction (500–1000 μm). The uneven structure of the TiO_2_ layer in the smaller fraction granules explained these results. The photocatalytic of TiO_2_-coated granules remained nearly the same after recycling by washing with ultrapure water and drying [75].

Al_2_O_3_ is a good substrate to coat with TiO_2_ to form TiO_2_/Al_2_O_3_ structures [76,77]. For example, Xu et al. (2009) prepared TiO_2_/Al_2_O_3_ microspheres using the sol–spray–calcination method. Briefly, powder TiO_2_ NPs (Degussa P25) were mixed with the Al_2_O_3_ powder in a TiO_2_/Al_2_O_3_ molar ratio of 50:1. Then, a spray layer was used to produce microspheres. The TiO_2_/Al_2_O_3_ microspheres, therefore, were calcined at 500 °C for 3 h. The obtained TiO_2_/Al_2_O_3_ microspheres had a diameter in the range of 20–100 μm with a surface area of 33.86 m^2^/g. The TiO_2_/Al_2_O_3_ microspheres showed good photocatalytic activity, whereby 80% of humic acid (HA) was degraded after 140 min. The photocatalytic activity of TiO_2_/Al_2_O_3_ microspheres remained at around 70% after 20 cycles of reuse [77].

TiO_2_ could also be coated on the porous activated carbon (AC) to form TiO_2_/AC photocatalysts. Arana et al. (2004) coated TiO_2_ NPs on the surface of activated carbon (AC) by mixing and stirring with activated carbon (7% *w*/*w*) for 1 h. The obtained TiO_2_/AC had a diameter of 6 μm. Compared with bareTiO_2_, TiO_2_/AC photocatalysts displayed almost no deactivation in any degradation experiments against gas-phase alcohols (methanol, ethanol, 1-propanol, and 1-butanol) [78]. In addition, Ouzzine et al. (2014) used a sol–gel method to coat TiO_2_ on the surface of spherical AC. The advantages of spherical activated carbon compared to the powdered and the granular activated carbon come from its smoother surface, better fluidity, and higher mechanical strength. The oxidation treatment at low temperatures is enough to obtain the TiO_2_/AC with high photocatalytic activity against propene at low concentration [79].

### 2.2. TiO_2_ Hollow Microscale Structures

TiO_2_ with hollow structures has many advantages, such as improved light scattering and slow photon effects, charge combination suppression, as well as a large number of reactive sites on the surfaces of the shells [21]. A solid structure with an empty side inside a distinct shell can be defined as a “hollow nanostructure”. According to Xiao et al. (2018), hollow-nanostructure TiO_2_ has enhanced photocatalytic activities due to the improvement of the harvesting of light energy via light scattering and slow photon effects, the suppression of charge separation by the decrease of charge transfer distance and separation of charge carriers, and the promotion of surface reactions due to a large accessible surface area [21].

**Figure 5:**
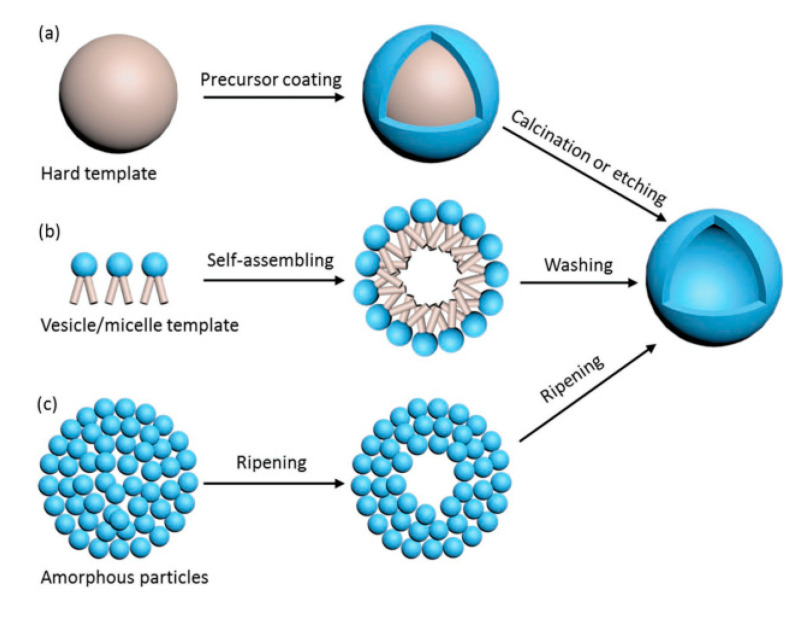
Preparation of TiO_2_ hollow spheres via the hard-templating method (**a**), soft-templating method (**b**), and self-templating method (**c**). Reprinted with permission from [21]. Copyright 2018, John Wiley and Sons.

There are different ways to synthesize TiO_2_ hollow sphere structures: the template-free method, the self-templating method, the soft-templating method, and the hard-templating method (Figure 5) [21]. The details of these synthesis methods, as well as the advantages and disadvantages of TiO_2_ hollow nanostructures, can be found in the outstanding review of Xiao et al. (2018) [21]. In the present review, we introduce only some remarkable examples of the preparation of hollow structures.

Briefly, in the hard-templating methods, the TiO_2_ precursor is coated outside the rigid template and the hollow structure is obtained after the calcination or etching process (Figure 5a). The limitation of these methods is due to the complexity of the template removal process (requiring calcination at high temperature or etching in alkaline and acid solutions) [21]. In soft-templating methods, the formation of hollow nanostructures is achieved via the difference of surface tension at the interfaces, such as water–oil or liquid–gas interfaces (Figure 5b). In most of these cases, removal of the soft template is not required. The limitations of the soft-templating methods derive from the lesser controllability of the shape, the shell thickness, and the size uniformity of the final products [21].

In contrast to the two above-noted synthesis strategies, the self-templating method has attracted interest due to its lower production cost and feasibility for scaling up to the industrial scale. Ostwald ripening is attributed in some papers to the mechanism of the growth of TiO_2_ spheres and hollow spheres [80,81]. Ostwald ripening is a thermodynamic process that is tailored by the differences in Gibbs energy (∆G) between the high G of the precursor and the low G of the resultant hollow nanostructure. A hollow nanostructure can be achieved by the formation of the shell, which is composed of large particles, while the core is left vacant (Figure 5c). Other remarkable principles entailed in the self-templating of hollow spheres are the Kirkendall effect, galvanic replacement, and surface-protected etching [82,83,84].

**Figure 6:**
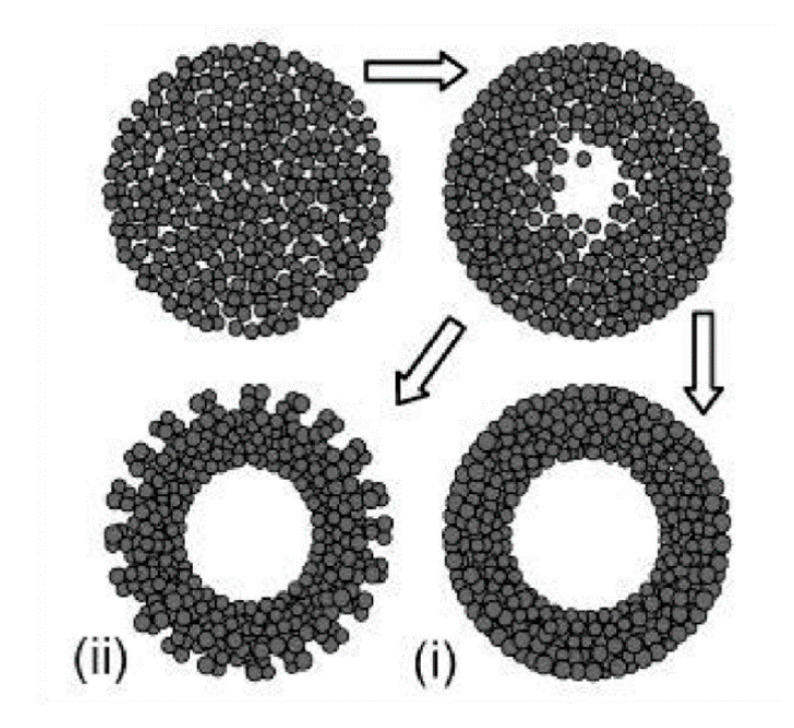
Two types of TiO_2_ hollow structures via Oswald ripening by hydrothermal process. Type (**i**) shows a dense and smooth surface, while type (**ii**) displays a less compact surface due to the achieved crystallite extrusion. Reprinted with permission from [80]. Copyright 2004, American Chemical Society.

Similarly to the case of TiO_2_ spheres, the hydrothermal technique can be used to prepare TiO_2_ hollow spheres via the self-templating approach. According to Yang et al. (2004), there are two basic types of hollow spheres: type (i) and type (ii) (Figure 6) [80].

Yang et al. (2004) prepared hollow anatase TiO_2_ spheres via Oswald ripening under hydrothermal conditions. TiO_2_ was prepared with titanium tetrafluoride (TiF_4_) as a precursor and the hydrothermal process was operated at 140–220 °C for 1.5–100 h. They observed that when the reaction time is short TiO_2_ spheres have a solid core, and that hollow spheres are observed when the reaction time is prolonged. The high concentration of TiF_4_ imparts a thicker shell due to the higher growth rate. Additionally, different additives have different effects on the growth of a hollow structure. For example, while thiourea can accelerate the hollowing process, the effect of urea is negligible. This can be explained by the difference of chelating abilities between: S = C and :O = C with respect to the titanium cations, as well as the difference in the chemical natures of their hydrolysis products. The obtained TiO_2_ hollow structure has a diameter in the range of 30–50 nm and lengths in the range of 150–250 nm. With 30 mL of TiF_4_ at 180 °C for 50 h of reaction time the TiO_2_ nanosphere type (i) can be obtained, while the TiO_2_ nanosphere type (ii) can be obtained with TiF_4_ (30 mL) + thiourea (10 mg) at 180 °C for 10 h. Yang et al. (2004) also concluded that a suitable temperature should be ≥ 160 °C. To obtain hollow TiO_2_ spheres, when the temperature reaches 220 °C, the reaction time can be reduced to 5 h [80]. Kang (2012) prepared mesoporous TiO_2_ hollow spheres with titanium butoxide (TB) as a precursor via a solvothermal process without the use of any templates or surfactants. The obtained hollow spheres had a specific surface area of 141 m^2^/g, a diameter of 700 nm, and a shell thickness of 90 nm. Their photocatalytic degradation of methyl orange (MO) was 98% after 30 min irradiation of UV light (300 W) [85]. Ma et al. (2019) found that TiO_2_ hollow spheres can be composed of different nanobuilding blocks by adjusting the starting solution. In detail, the presence of NH_3_●H_2_O could lead to TiO_2_ hollow microspheres composed of nanoparticles (THPs), the absence of NH_3_●OH could produce TiO_2_ hollow microspheres composed of mesoporous nanospheres (THSs), and the hydrothermal treatment in NaOH could result in TiO_2_ hollow microspheres composed of nanowires (THWs). The differences in the structure of TiO_2_ hollow microspheres could lead to the differences in photocatalytic performances. The THPs showed the lowest photocatalytic activity, while the THSs displayed the highest photocatalytic activity against Rhodamine B (RhB) in the same conditions. The advantages of THPs could be explained by its highest surface areas [48]. Recently, Xie et al. (2019) applied solvothermal to prepare SnO_2_/TiO_2_ microspheres. The obtained SnO_2_/TiO_2_ microspheres continued to anneal at 450 °C for 2 h. In the results, microspheres with diameters in the range of 500–1000 nm were assembled with a surface area of 199.3 m^2^/g. SnO_2_/TiO_2_ granules were then utilized as a scattering layer for dye-sensitized solar cells, showing 28.1% improvement of the photovoltaic conversion efficiency when compared with bare nanocrystalline-based cells [86]. The TiO_2_ microsphere was also coated with noble metal to improve its photocatalytic performances. Chowhury et al. (2019) decorated gold (Au) nanoparticles on TiO_2_ microspheres to degrade phenol under visible light irradiation. They found that the TiO_2_ microspheres with 5 wt% Au showed the highest photocatalytic performances, whereby 97% of phenol was removed after irradiation for 1 h by visible light [87].

In comparison with the conventional methods, which localize overheating output from the hot surface of the reaction vessels, possibly leading to product composition changes in cases of heating for elongated periods, the microwave method takes advantage of the potential to produce uniform internal heating by direct coupling of microwave energy with the polar molecules present in the reaction mixture [81]. In order to produce TiO_2_ hollow spheres, Alosfur et al. (2018) recently utilized a 100 mL solution of titanium (IV) isopropoxide (TTIP 0.2 M; 95% ethanol) placed in a microwave oven with a reflux device and magnetic stirrers at 550 W for 5 min. Then, the precipitate was centrifuged (4000 rpm for 5 min), dried at 90 °C in air overnight, and calcined at 500 °C for 1 h to obtain anatase TiO_2_ hollow spheres. The resultant spheres had sizes in the range of 200 to 500 nm, with pore sizes in the range of 2–50 nm and a surface area of 172.3 m^2^/g. The growth mechanism of the TiO_2_ hollow spheres was attributed to the Ostwald ripening process during heating. The photocatalytic activity of the TiO_2_ hollow spheres for MB was high under both UV and visible light irradiation; this was attributed to the organization of the NPs into a hierarchical structure that can prevent random aggregation [81].

Recently, Balati et al. (2020) used pulsed laser ablation (PLAL) to prepare hydrogenated anatase- and rutile-based inorganic hollow microspheres (HBTiO_2_/RBIHM). Thus, HBTiO_2_/RBIHM was decorated with MoS_2_ nanosheets (HBTiO_2_/RBIHM-MoS_2_) by microwave irradiation for the visible light arsenic photooxidation. The interconnected layers of MoS_2_ resulted in the formation of porous 3D nanostructures in HBTiO_2_/RBIHM-MoS_2_. HBTiO_2_/RBIHM-MoS_2_ could achieve 96.6% arsenite photooxidation efficiency, and 70.3% and 5200 μg/g arsenate adsorption capacity. The synergetic effects from RBIHM-HBTiO_2_, RBIHM-MoS_2_, and HBTiO_2_/RBIHM heterojunctions explained the performance of HBTiO_2_/RBIHM-MoS_2_ [88]. 

The soft-templating method is also a popular method used for the preparation of TiO_2_ hollow spheres. Similar to TiO_2_ solid spheres, different surfactants can be used to synthesize mesoporous TiO_2_ spheres with particle sizes ranging from submicrometers to micrometers using dodecyl-amine as a surfactant [43]. In the study by Ren et al. (2003), TiO_2_ hollow microspheres were synthesized with poly(ethylene oxide) as a surfactant. The obtained hollow microspheres had a surface area of 0.378 m^2^/g, a pore volume of 0.34 cm^3^/g, and a pore size of 2.6 nm. The TiO_2_ hollow microsphere formation could be explained as follows: the hydrolyzed alkoxides (nanosized Ti-O particles) interacted with amphiphilic surfactant molecules via weak hydrogen bonding, forming mesostructured hybrid inorganic–organic precursory NPs, then gelling to form an -O-Ti-O-Ti- network under autoclaving by polycondensation between NP precursors, finally leading to mesostructured spherical shells. However, the authors stated that the obtained TiO_2_ hollow microspheres had an irregular shape due to the lack of complete hydrolysis, and thus could be destroyed in the calcination process. Due to the incomplete condensation, there was a large number of hydroxyl groups on the surfaces of the TiO_2_ microspheres. These hydroxyl groups could function as active sites in catalysis or as binding sites for further surface modification [89].

Zhang et al. (2005) used the micelles of salicylic acid (SA) anions and anilinium cations containing TiO_2_ for formation of polyaniline (PANI)/TiO_2_ microspheres. PANI has been widely applied in the preparation of TiO_2_ hollow spheres due to its cheap, simple preparation, uniquely controllable properties via oxidation and protonation states, outstanding environmental stability, and potential application to electronic devices [90]. The molar ratios of aniline (ANI) to SA and ammonium per-sulfate ((NH_4_)_2_S_2_O_8_), APS) were both 1:1. The PANI-SA microsphere was formed by the hydrogen bond between the -OH group of SA and the amine group of PANI. The PANI/TiO_2_ microsphere was believed to have a core–shell structure with TiO_2_ as the “core” due to its hydrophobicity and ANI/SA as the “shell” due to the hydrophilicity of the SA dopant (-COOH groups). The polymerization process was expected to occur at the interface of micelle–water due to the hydrophobicity of APS as an oxidant, while the growth of the microspheres was managed by the accretion process [91,92,93,94]. The obtained polyaniline/TiO_2_ microspheres (PANI-SA/TiO_2_) had an average diameter of around 2.5–3.6 µm, while the thickness of the TiO_2_ layer was ~15 nm. The TiO_2_ NPs’ position in the composite could be confirmed by water contact angle measurements. TiO_2_ NPs are hydrophobic, and so the water contact angle of PANI-SA/TiO_2_ was increased to 57.5°, while this number for bare PANI-SA/TiO_2_ was 41.2°. However, the water contact angle of PANI-SA/TiO_2_ was still lower than that of PANI-β-NSA/TiO_2_ nanotubes (β-NSA: β-naphthalenesulfonic acid) prepared by Zhang et al. (2003) in another study. The lower contact angle of PANI-SA/TiO_2_ indicated that most of the TiO_2_ NPs were filled in the hollow interior of the PANI-SA microspheres [90].

In addition, Zhang and Wan (2003) prepared polyaniline/TiO_2_ (PANI-TiO_2_) composite nanotubes with diameters in the range of 90–130 nm in the presence of β-naphthalenesulfonic acid (β-NSA). They observed that the morphology of the polyaniline-β-NSA/TiO_2_ (PANI-β-NSA/TiO_2_) composite was influenced by the TiO_2_ concentration. When the TiO_2_ concentration was lower than 0.08 M, the PANI-β-NSA/TiO_2_ composites formed fibers; but when the concentration was 0.12 M, the morphology of PANI-β-NSA/TiO_2_ composites was changed to the granule form. The “core–shell” structure of PANI-β-NSA/TiO_2_ was similar to that of PANI-SA/TiO_2_ above. However, energy-dispersive X-ray data showed that the TiO_2_ NPs were on the walls of the PANI-β-NSA/TiO_2_ nanotubes rather than inside of them. This phenomenon was confirmed by the PANI-β-NSA/TiO_2_ hydrophobicity (water contact angle: 98.5°) [95].

Gelatin-filled reverse emulsion can also be applied to the preparation of TiO_2_ hollow spheres on the nanoscale with water as the polar phase, *n*-dodecane as the non-polar phase, titanium tetrachloride (TiCl_4_) as a precursor, cetyltrimethylammonium bromide (CTAB) as the surfactant, and 1-hexanol as the co-surfactant. The obtained hollow structures showed an outer diameter of 25–35 nm and a wall thickness of 15–20 nm. Although the TiO_2_ hollow spheres were covered by gelatin, their photocatalytic activity was nonetheless similar to that of TiO_2_ powder (P25) in their removal of MB (pH = 8) under visible light irradiation [96].

In the hard-templating method, different polymers can be used as the hard template. For example, Wang et al. (2002) prepared hollow shells via the layer-by-layer self-assembly strategy with exfoliated unilamellar titania nanosheets used as inorganic shell building blocks. Spheres of polystyrene (PS) and poly(methyl methacrylate) (PMMA) were used as colloidal templates, and while adjusting the surface charge of these spheres, polyethylenimine was applied. The TiO_2_ shell thickness could be adjusted by coating cycles. The polymer core was removed via the calcination process at 500 °C or by UV-irradiation, thereby obtaining titania hollow shells with a smooth surface and small thickness (~5 nm). UV irradiation is a “green” technique by which low temperature is applied to remove the polymer template [97,98]. Interestingly, different treatments, therefore, lead to differences in the optical properties of titania hollow spheres. In another study, the ultraviolet-visible (UV-vis) spectra of calcined hollow spheres were red-shifted compared to UV-irradiated hollow spheres. The transformation of nanosheets with a molecular thickness to the anatase phase could explain the visible differences [99].

Syoufian et al. (2007) applied sulfonated PS latex particles as a hard template in order to prepare submicrometer-sized titania hollow spheres. Titania-PS composites were calcined at 400 °C to remove the template and form TiO_2_ hollow spheres. The authors found that the low titanium butoxide (TB) concentration could lead to the formation of hollow spheres with a fragile shell. In contrast, the high TB concentration could result in a relatively smooth and rigid shell. Additionally, when the TB concentrations were 0.15, 0.20, 0.25, and 0.50 M, the shell thicknesses of the titania hollow spheres were 9, 14, 17, and 23 nm, respectively, while their void sizes were 147, 151, 155 and 159 nm, respectively [100]. Hollow spheres synthesized from 0.20 M of TB have the highest anatase phase. In another study, this time on the photodecomposition of MB under the irradiation of a 150 W xenon lamp, the reaction rate was increased two times by the injection of peroxydisulfate as an electron scavenger at an optimal concentration (10 mM). The enhancements of the photocatalytic reaction by electron acceptors can be explained in different ways: (i) the prevention of the recombination of electron–hole pairs by acceptance of the electron from the conduction band, (ii) the increase of the concentration of hydroxyl radical (●OH), and (iii) the generation of other oxidizing species (e.g., ●SO_4_^−^) to promote the intermediate compound oxidation rate [101,102,103]. Further increase of peroxydisulfate leads to saturation of the reaction rate due to excess amounts of SO_4_^2−^ species [73].

SiO_2_ can also be a candidate for the hard-templating method. One strategy to shift the TiO_2_ photocatalytic activity towards the visible region is the combination with narrower bandgap semiconductors, such as cadmium sulfide (CdS) [104,105]. Sue et al. (2014) used the sonochemical method to synthesize a CdS-TiO_2_ hollow structure. First, a TiO_2_ nanolayer was coated on the surface of SiO_2_ via the hydrolysis of TB in DI water under vigorous stirring. Then, CdS was deposited on the SiO_2_-TiO_2_ core–shell structure via the sonochemical process. SiO_2_ was removed via the addition of NaOH, thus forming the CdS-TiO_2_ hollow structure. The obtained structures have an average diameter of 300 nm, with the thickness of the TiO_2_ shells being about 30 nm, and the diameter of the voids being about 237 nm. Compared with the pure TiO_2_ samples, the UV-vis diffuse spectra of the CdS-TiO_2_ hollow structure was shifted to the visible light region. With rhodamine B (RhB), >90% of pollutants was removed by the CdS-TiO_2_ hollow structure after 120 min under visible light irradiation, more so than other samples, such as P25, CdS, and SiO_2_-TiO_2_. However, after three cycles of the photocatalytic reaction, the degradation efficiency of the CdS-TiO_2_ hollow structure was reduced to ~30%. This reduction was attributed to the photocorrosion of CdS, as well as the mass loss of the catalyst [106].

Besides the above strategies, TiO_2_ hollow microspheres can be prepared by spray-drying of an exfoliated titanate sheet suspension without the assistance of any templates. Afterward, the spray-dried gel is calcined at 650 °C/h to destroy the lamellar structure and promote the growth of TiO_2_ anatase. The obtained TiO_2_ hollow spheres are 10 to 50 μm in size and have a shell thickness of 0.1 μm [107].

There are some differences between TiO_2_ dense microscale structures and TiO_2_ hollow microscale structures. For the same diameter, titania hollow spheres have a lower density and larger surface area compared with TiO_2_ dense spheres [108]. Additionally, compared with TiO_2_ dense spheres, the UV absorption spectra of hollow spheres show redshift [109]. This redshift could come from oxygen defects during the formation of TiO_2_ particles or by the doping of C or S atoms into TiO_2_ particles [109,110,111,112,113].

In both TiO_2_ solid and hollow microscale structures, there are still many challenges that remain, notwithstanding their advantages. Firstly, the photocatalytic mechanism of these materials is not fully understood. Secondly, the complexity of synthesis methods also prevents industrial applications of TiO_2_ solid and hollow spheres. Besides, the fabrication of these materials with both high crystallinity and large surface areas is still a major challenge. For TiO_2_ hollow spheres, the effects of morphological variations of shape, diameter, shell thickness, and numbers of shells should be further investigated [21].

## 3. TiO_2_ Macroscale Structures

### 3.1. Pure TiO_2_ Pellets

The advantages of TiO_2_ pellets come from their ease of production, low required amounts of raw materials, lack of substrate necessity, and compatibility with mass production [20,51]. Compared with the above microscale TiO_2_ pellets, TiO_2_ pellets can be prepared by different and simple conventional methods, such as tumble growth, tableting, and extrusion. However, these methods have some limitations, such as poor mechanical strength and low attrition resistance [53].

Dervos et al. (2004) pressed powder uniaxially in a hydraulic press at 250 psi into pellets measuring 10 mm in diameter and 3 mm in thickness. The pellets were calcinated at different temperatures (850, 1050, 1150, and 1180 °C) for 2 h. The results showed that at 915 °C, the anatase crystal structure was converted into the rutile structure. TiO_2_ pellets, which were calcinated at 1180 °C, had high packing microcrystal density [114]. Yao et al. (2009) also prepared TiO_2_ ceramic pellets from TiO_2_ powder. Briefly, TiO_2_ powders of 7–8 and 20–50 nm diameters were mixed with DI water (25–30 wt%) and kneaded into TiO_2_ ceramic pellets (diameter: 2–4 nm). Then, ceramic pellets were dried and calcined at different times and temperatures. It was observed that the ceramic pellets from the 20–50 nm TiO_2_ powder were unstable and easily broken down when immersed into aqueous solution. The obtained XRD patterns showed that the TiO_2_ ceramic pellets were similar to TiO_2_ powder. Still, wider diffraction peaks were observed, indicating that the small-size quantum effects were more significant, the grain size was smaller, and the surface activity was higher. After the calcination process, the crystallization defects of the ceramic pellets were reduced. TiO_2_ ceramic pellets are structured with array stacking of NPs and possess a porous structure with high porosity, irregular pores, and rarely closed pores. Compared with titanium sol and silica sol, water is the better adhesive, with which the TiO_2_ ceramic pellets showed better sterilization performance. The antimicrobial activity against *Colibacillus* was 99% after 3 h of photocatalytic reaction. The photocatalytic activity of porous TiO_2_ ceramic pellets did not change after their regeneration by calcination at 600 °C for 2 h [51].

Ultrasound has been used for wastewater treatment [115,116,117]. In ultrasound techniques, very high temperatures (up to several thousand Kelvin) and pressures (up to several hundred atmospheres) can be produced by cavitation in the collapse of gas bubbles in aqueous solution, resulting in the thermal division of water molecules into H atoms and ●OH [118,119]. The combination of photocatalytic and ultrasonic irradiation could lead to an increase in pollutant degradation due to the increase of ●OH. Additionally, the existence of a heterogeneous catalyst could increase the formation rate of cavitation bubbles by supplying additional nuclei [120,121,122], which would accelerate the thermal dissociation of H_2_O molecules and the ●OH formation. Shimizu et al. (2007) studied MB degradation by ultrasound irradiation (60 min, frequency: 39 kHz, emission power: 200 W) of TiO_2_ pellets (2.0 mm) in aqueous solution. A significant reduction in the MB concentration was achieved after 60 min. With the addition of H_2_O_2_, the MB degradation was increased from 22 to 85%. By contrast, the H_2_O_2_ addition did not affect the removal of MB when Al_2_O_3_ was present in the system. Dimethyl sulfoxide (DMSO), methanol, and mannitol were used to investigate the influence of radical scavenging agents on the removal of MB. The results showed that DMSO was the most effective scavenging agent. The optimal pH for the removal process was 7 [119].

**Figure 7:**
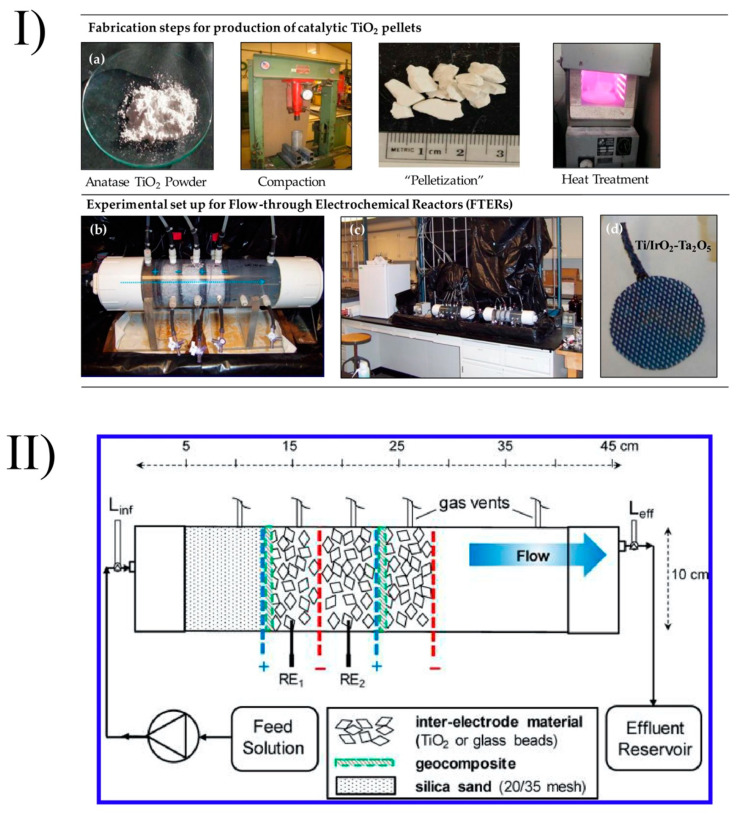
Fabrication steps for production of catalytic TiO_2_ pellets (**I**) and scheme of the flow-through electrochemical reactor (**II**). RE are reference electrodes. Reprinted with permission from [123]. Copyright 2016, American Chemical Society.

Recently, Jasmann et al. (2016) prepared TiO_2_ pellets of between 3.3 and 9.42 mm in mesh size via conventional compacting that had been prepared and embedded into a flow-through electrochemical reactor for the removal of 1,4-dioxane via advanced electrochemical oxidation. After compacting, the pellets were calcined at 500–1000 °C for 4 h (Figure 7). They found that TiO_2_ pellets sintered at 500–800 °C were friable and readily subject to abrasion. However, TiO_2_ pellets sintered at a higher temperature (880–1000 °C) had high mechanical strength. The sintering temperature affected the crystallinity of TiO_2_ pellets. For example, TiO_2_ pellets calcined at 700 and 880 °C contained 98% anatase and 2% rutile, while these numbers for TiO_2_ calcined at 1000 °C were 14% and 86%, respectively. They showed that the TiO_2_ pellets had high removal efficiency (>97%)—4.6 times higher than the non-catalyzed electrolytic reactor. It was found that TiO_2_ could be activated in the dark even when the electrodes were electrically insulated. The most significant advantages of these TiO_2_ pellets came from their high catalytic activity in low-ionic-strength water, where conventional electrochemical processes commonly fail [123].

In the production of ceramic materials, pressing for densification is the most important step. There are different pressing methods, such as conventional uniaxial pressing, cold isostatic pressing, and hot isostatic pressing. Among them, hot isostatic pressing (HIPing) is considered to be an advanced technique due to the volumetric uniformity in the pressure make-up and continuous densification [124,125]. Application of high-pressure during heating, which leads to high densification, is the most important reason to choose hot isostatic pressing for preparation of TiO_2_ pellets. Additionally, phase stability and transformation in oxide ceramics can be altered under an argon atmosphere [125]. Importantly, both temperature and pressure influence the phase transition from the anatase phase to the rutile phase during the sintering process [126,127].

As mentioned in the Introduction, TiO_2_ has three different polymorphic structures, which are anatase, rutile, and brookite. Among them, rutile is the thermodynamically stable phase and is the one mostly utilized in optical applications. In contrast, anatase is a metastable phase, is hydrophilic, and has higher photocatalytic activity [11]. However, as mentioned above, many studies have claimed that the mixture of anatase and rutile at a suitable ratio has a higher photocatalytic activity than only anatase or rutile in the structure of material [11,12,13,14,15]. Erol and Ertugul (2018) investigated the influence of the heating method and temperature on the physical, structural, and photocatalytic performances of TiO_2_ pellets produced by either conventional heating (CH) or hot isostatic pressing (HIPing). The pellets of submicron TiO_2_ powders were then calcined at 600, 650, 700, 750, and 1000 °C in order to compare the densification behaviors and the transformation between the two methods. In the HIPing method, an HIPing furnace was used with graphite heating elements under an argon atmosphere and 15,000 Psi pressure. One advantage of HIPing relative to CH was found to come from its shorter cycle duration. The average heating rates were 8 °C/min and 12 °C/min for HIPing and CH, respectively. The authors found that the transformation from anatase to rutile by conventional heating was slightly faster, and that densification was higher for lower temperatures, while HIPing showed high densification above 750 °C, as it also delayed rutile transformation. The lower porosity and higher densification of the CH samples heated at 650, 700, and 750 °C relative to the HIPing samples could be explained by the degree of carbon contamination during the process, which would have delayed the phase transformation and inhibited densification. The carbon contamination could be explained by the graphite heating elements in HIPing. Because of the driving forces of temperature and pressure, some amount of carbon contamination could effect a change of TiO_2_ pellet structure [128,129,130]. Additionally, the phase transformation differences between the two methods come from the difference in the applied pressure and atmosphere. In addition, results have shown that HIPing samples with mixed-phase structures display the highest photocatalytic activity. In detail, the Langmuir–Hinshelwood (L-H) kinetics model can be used to explain the heterogeneous catalytic processes. For both methods, the highest photocatalytic performance is displayed at 700 °C. The best sample involved HIPing at 700 °C, which had a ratio of anatase-to-rutile of close to 1:1. Additionally, carbon contamination lowers the optical bandgap and thereby improves the photocatalytic performance of TiO_2_ [1].

**Figure 8:**
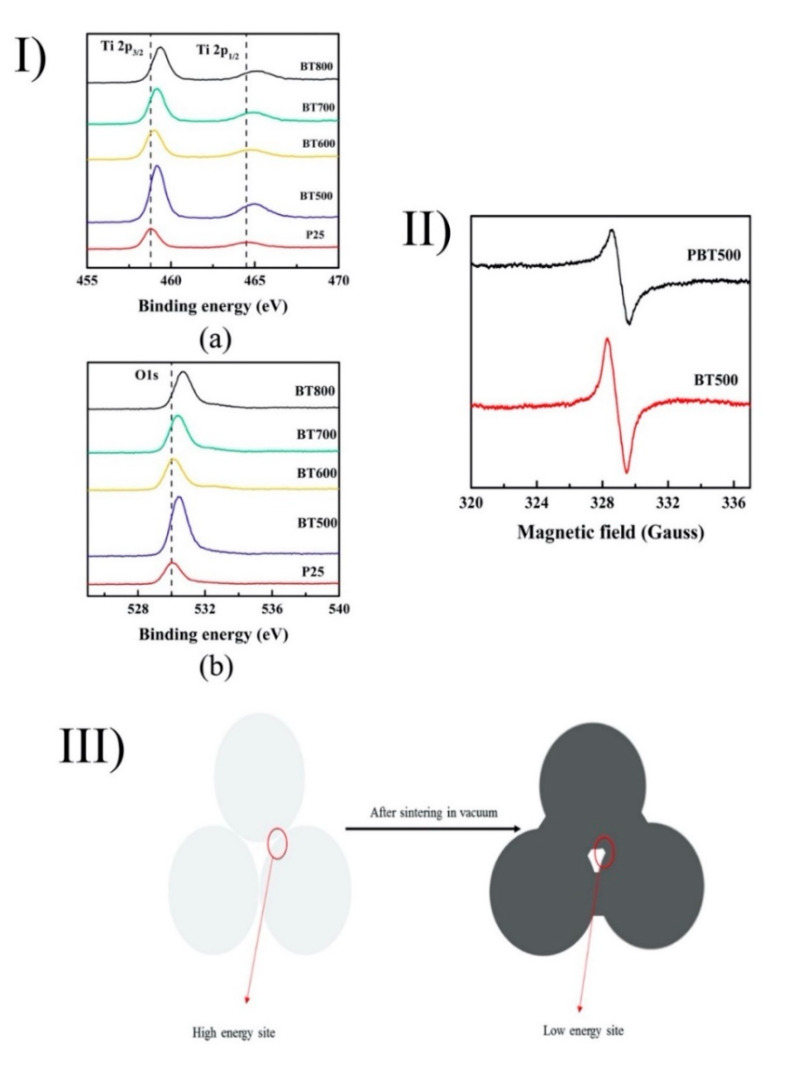
(**I**) XPS spectra of Ti 2p (**a**) and O 1s (**b**) of P25 and black TiO_2_ samples. (**II**) Electron paramagnetic resonance (EPR) spectra of compact pellets sintering at 500 °C (BT500) and P25 powder sintering at 500 °C (PBT500) under vacuum condition. (**III**) Scheme of formation of low-energy sites via sintering in a vacuum. Reprinted with permission from [32]. Copyright 2018, American Chemical Society.

Black TiO_2_, which was first introduced by Chen et al. [131], has recently attracted attention due to its outstanding photocatalytic activity under visible light. The common method to prepare black TiO_2_ is thermal treatment under a hydrogen atmosphere. However, working with hydrogen is dangerous. Due to its flammability, hydrogen can immediately explode when it interacts with oxygen. Katal et al. (2018) synthesized black TiO_2_ by sintering P25 pellets under a vacuum atmosphere at various temperatures (500–800 °C). In their observations, the high sintering temperatures transferred the TiO_2_ phase from anatase to rutile, similarly to other reports in the literature [132,133]. Additionally, the increase of the sintering temperatures increased the crystal size, while the surface area, porosity, and pore volume were decreased. The pellets were prepared using the conventional processing technique at ambient temperature. The differences between sintered powder and pellets via the formation of oxygen vacancy density and changes of color were also investigated. In the results, P25 powder changed to a darker color but was not completely black, as with the P25 pellets. The loss of oxygen and the oxygen vacancy formation could explain the change of color from white to black [54,134]. Importantly, with the increase in the sintering temperature, the energy bandgap decreased. Compared with P25, the red-shift absorbance spectra in the bandgap were achieved in the case of the black TiO_2_ pellets. From the XPS spectra, the stronger shoulder peaks in the higher binding energy of the O 1s peaks of the black TiO_2_ samples could explain the formation of the oxygen vacancy (Figure 8) [135]. Under a hydrogen atmosphere, the formation of the oxygen vacancy (V_o_) was explained by the following reaction:

H_2_ + O_L_ → H_2_O + V_o_ + e^−^

where O_L_ is the oxygen in the lattice of TiO_2_.

However, under a vacuum atmosphere, the oxygen vacancy could be formed by the following reaction:

O_L_ = ½ O_2_(g) + V_o_ + e^−^

In addition, the oxygen vacancy in the black TiO_2_ pellets was higher than the powder form, as confirmed by EPR (Figure 8). Figure 8 shows that the sintering process leads to an interface region between the low-energy site and particle changes (neck region) as a result of compaction. These low-energy sites, thus, may lead to the decease of the O_2_^−^ adsorption sites, which is beneficial for the formation of the diamagnetic oxygen species. Additionally, the decrease of the energy bandgap could explain the photocatalytic performances of black TiO_2_ under visible light irradiation. With regard to photocatalytic activity against acetaminophen (ACE), the as-prepared samples showed similar performances to black TiO_2_ synthesized under the hydrogen atmosphere. In contrast, the P25 pellets sintered at 500 °C showed the best photocatalytic performances under AM 1.5 G solar light illumination. The presence of the oxygen vacancy was maintained, even after 6 months. Additionally, the stability of black TiO_2_ pellets was acceptable in both the short (one month) and the long term (six months) [32].

Floating photocatalysts confer many remarkable benefits due to the optimal light illumination process, especially for a system using solar irradiation, while the oxygenation process could be maximized according to the closeness to the air–water interface, especially for a non-stirring system. These properties, thus, lead to increases in both the formation rate of radicals and oxidation efficiency [69]. Due to the different highlighted characteristics, such as the light weight, large surface area, high specific strength, and exceptional permeability, porous TiO_2_ ceramics are usable for pollutant transfer and diffusion [136]. Freeze-drying is a simple, low-cost, and eco-friendly synthetic technique for the fabrication of porous ceramics. Compared with conventional techniques, freeze-drying is a better way to adjust pore distribution [137,138,139,140]. The formation of three-dimensional interconnected pore channels can be obtained when the frozen vehicle network exists in the body of ceramics. The pore structure and pore size of ceramics can be easily modified by adjusting the parameters of free-drying processes [141]. H_2_O and *tert*-butanol can be used as frozen vehicles to produce porous ceramics. In order to eliminate the expensive sub-0 °C freezing process, camphene can be used as a novel frozen vehicle [142].

Xing et al. (2014) prepared floating TiO_2_ ceramics via a camphene-based freeze-drying route for photocatalytic degradation of a micropollutant pesticide. The results showed that the resultant ethanediamine (EN)-modified TiO_2_ ceramics can prevent the growth of undesirable TiO_2_ particles, as well as the transformation of the anatase to the rutile phase, even at 800 °C. This was explained by the interaction and binding of EN on the surface of Degussa P25 [143]. The BET surface area of TiO_2_ was maintained by the EN modification. With the rise of the TiO_2_ solid content from 10 to 20 wt%, the porosity was decreased from 95.2 to 89.6%, while the compressive strength was increased from 0.59 to 0.98 MPa. The TiO_2_ ceramic with an original content of ~15 wt% had the optimal compressive strength and porosity. In the photodegradation of atrazine and thiobencarb, the TOC removal efficiencies were as high as 95.7 and 96.7%, respectively. These efficiencies could be maintained for up to 6 cycles with no obvious changes [34].

**Figure 9:**
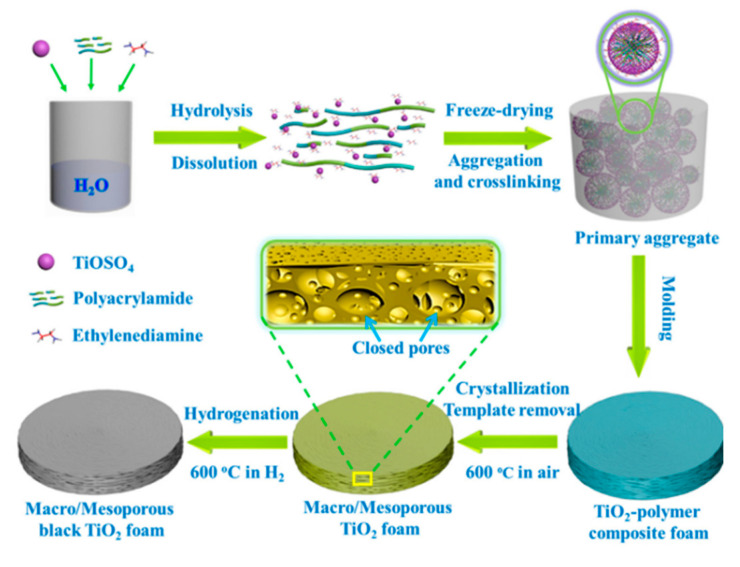
Scheme of the formation of self-floating amphiphilic macro/mesoporous black TiO_2_ foams. Reprinted with permission from [54]. Copyright 2017, Elsevier.

Recently, Zhang et al. (2017) prepared self-floating amphiphilic black TiO_2_ via a freeze-drying method combined with cast-molding technology and sintering at high temperature under a hydrogen atmosphere. In their study, ethylenediamine was used as an acid–base equilibrium agent, as well as to prevent the collapse of 3D macro-mesoporous networks. The presence of ethylenediamine also prevented the transformation of anatase to rutile and the growth of undesirable particles under hydrogen atmospheres at 600 °C (Figure 9). The obtained black TiO_2_ foams could easily float on the surface of the water, and their photocatalytic activity could be shifted to the visible light region. The photocatalytic activity of the self-floating amphiphilic black TiO_2_ foams was seven times higher than that of commercial Degussa P25 under a 300 W Xenon lamp with air mass (AM) 1.5 filters. The 3D macro-mesoporous networks, which are beneficial for mass transport; the super amphiphilicity, which is beneficial for rapid adsorption; the floating ability; and the presence of Ti^3+^ in the frameworks explained the high photocatalytic capacity of the black TiO_2_ foams [54].

### 3.2. TiO_2_ Composite Pellets

Besides pure TiO_2_ pellets, TiO_2_ composite pellets have been used as well. Although TiO_2_ pellets have proved to the better than TiO_2_ powder in gas-phase applications [144,145], the activity of TiO_2_ was lost because of both the pelletization process and the drying step [146]. The loss of photocatalytic activity can be partially recovered via pelletization due to the existence of carbon materials. The pelletization of TiO_2_ with the existence of carbon materials has been shown in the literature [147,148,149,150,151]. The improvement of photocatalytic activity could come from the condensation of organic molecules on the carbon surface [148]. In an aqueous solution, according to the existence of activated carbon, the acid–base characteristics of TiO_2_ have been changed [152]. Lillo-Rodenas et al. (2006) prepared a series of TiO_2_/C pellets using activated carbon, activated carbon fibers, carbon nanofibers, single-wall carbon nanotubes, multiwalled carbon nanotubes, expanded graphite, and carbon black. Relative to 100% TiO_2_ pellets, the TiO_2_/C pellets showed higher photocatalytic performance [146].

Bouazza et al. (2008) showed that besides carbon-based materials, composite TiO_2_ pellets with white additives, such as MCM-41, zeolites, metal-organic framework, SiO_2_, Al_2_O_3_, glass wool, and quartz wool, have higher photocatalytic performance in terms of propene degradation than 100% TiO_2_ pellets. A homogeneous mixture of TiO_2_ (0.7 g), M (chosen additive) (0.3 g) and DI water (1 mL) was extruded by a plastic syringe (5 mL) to form TiO_2_/M pellets. These pellets (~1 mm of diameter) were then cut into pieces measuring 10 mm in length and dried at 383 K for 12 h. TiO_2_/M pellets have variable specific surface areas, from very high (MCM-41, 1000 m^2^/g) to almost zero, such as for glass wool and quartz wool. In the results, neither the addition of different additives nor the pelletization process changed either the original crystalline composition or the crystalline sizes of the P25 powder. However, according to transmission electron microscopy (TEM) images, the dispersion of TiO_2_ was changed depending on the chosen additive. For example, TiO_2_/M1 showed high dispersion, while the dispersion of TiO_2_/M6 and TiO_2_/M8 was poorer. The authors also found that the flow rate can affect the photocatalytic ability of TiO_2_ pellets. For example, under the irradiation of UV light at 257.7 nm, complete oxidation could be obtained for most of the photocatalysts; however, when the flows were increased up to 30 and 60 mL/min, the photocatalytic activity was decreased. A similar phenomenon was obtained in the case of 365 nm irradiation. Surprisingly, Bouazza et al. (2008) made no recommendation for porosity or any mesoporous additives for maximization of photocatalytic activity. They claimed that neither the low electrical conductivity of TiO_2_/M pellets nor the UV-absorption spectra could be used to describe the high or low photocatalytic conversion of propene. Even the understanding of the differences in the photocatalytic activity of TiO_2_/M was not clear; they assumed that the variation in the hole-electron recombination characterizations could be used to explain these differences. Compared with the best TiO_2_/C pellets (TiO_2_/C1), their TiO_2_/M1 sample showed better photocatalytic performance, although C1 is larger than M1 in terms of both porosity and electric conductivity. Therefore, the authors concluded that the addition of white additives can recover the activity loss of TiO_2_ better after pelletization by introduction of carbon materials. Additionally, the activity of the TiO_2_/M pellets was retained after several cycles. Besides, no intermediate oxidation compounds were observed after finishing the propene oxidation [153].

Other than strategies such as noble metal loading, ion doping, and metal ion-implantation, binary metal oxides can be used to shift the wavelength range of TiO_2_ towards the visible region [52]. For example, Pal et al. (1999) showed that TiO_2_/Fe_2_O_3_ mixed oxides prepared via the sol–gel impregnation method had excellent absorption (570–600 nm) in the visible spectral region [154]. At the laboratory scale, magnetic α-Fe_2_O_3_ and γ-Fe_2_O_3_ can be recovered via magnetic separation. However, at the industrial scale, it is difficult to apply a magnetic force to isolate and recover photocatalysts from an aqueous solution system [52]. To overcome this limitation, Li et al. (2015) developed Fe_2_O_3_/TiO_2_ composite ceramics with 45 wt% of Fe_2_O_3_ for water treatment. The sintering temperature effects on the crystalline phase, physical characteristics, and photocatalytic activities of the composite pellets were evaluated. With the increase of sintering temperature, TiO_2_ was transformed from the anatase to the rutile phase and reacted with α-Fe_2_O_3_ to produce pseudo-brookite Fe_2_TiO_5_. However, above the 800 °C sintering temperature, only rutile TiO_2_ and Fe_2_TiO_5_ were obtained. In general, TiO_2_ can be converted from the anatase to the rutile phase with iron as the catalyst. Fe_2_TiO_5_ was present via the bulk reaction between α-Fe_2_O_3_ and rutile TiO_2_. The porosity and the photocatalytic performance of the Fe_2_O_3_/TiO_2_ composite pellets were reduced when the sintering temperature was increased, especially when it reached 1000 °C. This phenomenon explained the decrease in the photocatalytic properties of the composite ceramics. In general, the Fe_2_O_3_/TiO_2_ ceramics sintered at 880 °C (FTC-880) showed high photocatalytic activity for the removal of MB under both UV and visible light. Even in the third cycle, this composite sample still displayed a high decomposition rate (78% vs. 88% when first used under visible light, MB = 25 mg/L, pH = 4). The reduction of the photocatalytic activity of the composite ceramics was explained by the intermediate catalytic products on the catalyst surfaces. From scanning electron microscope (SEM) images, FTC-880 samples were formulated of the plate-like and rod-like structure. Besides, the FTC-880 sample displayed strong absorption in both the UV region (<400 nm) and the visible light (400–700 nm) regions. In addition, it showed high compressive strength (11 × 10^3^ kN/m^2^). However, the Fe_2_O_3_/TiO_2_ nanopowder still had higher photocatalytic performance compared with the Fe_2_O_3_/TiO_2_ ceramics. These results could be attributed to the higher surface area and the main TiO_2_ anatase of the Fe_2_O_3_/TiO_2_ nanopowder relative to the Fe_2_O_3_/TiO_2_ ceramics [52].

### 3.3. Immobilized TiO_2_ Macroscale Structure

Commercial P25 powder has been immobilized on different substrates, such as small glass spheres or beads, to improve its potential in wastewater treatment. A mixture of sol–gel TiO_2_ and TiO_2_-P25 immobilized on glass spheres via the dip-coating method showed excellent treatment performance for the removal of contaminants and pesticides in a pilot compound parabolic concentrator (CPC)-type reactor. Additionally, the immobilization of P25 on glass beads via the heat attachment method was successfully applied to degrade dyes and pharmaceuticals under UV radiation instead of sunlight [24]. In that same study, different regeneration methods were evaluated, such as chemicals (single or combination of HNO_3_, NaOH, NH_4_OH, and H_2_O_2_, with the assistance of UV irradiation) and water washing; UV exposure with pure air; high-humidity conditions for air-pollutant treatment; sonication treatment with water and methanol; and thermal processes.

**Figure 10:**
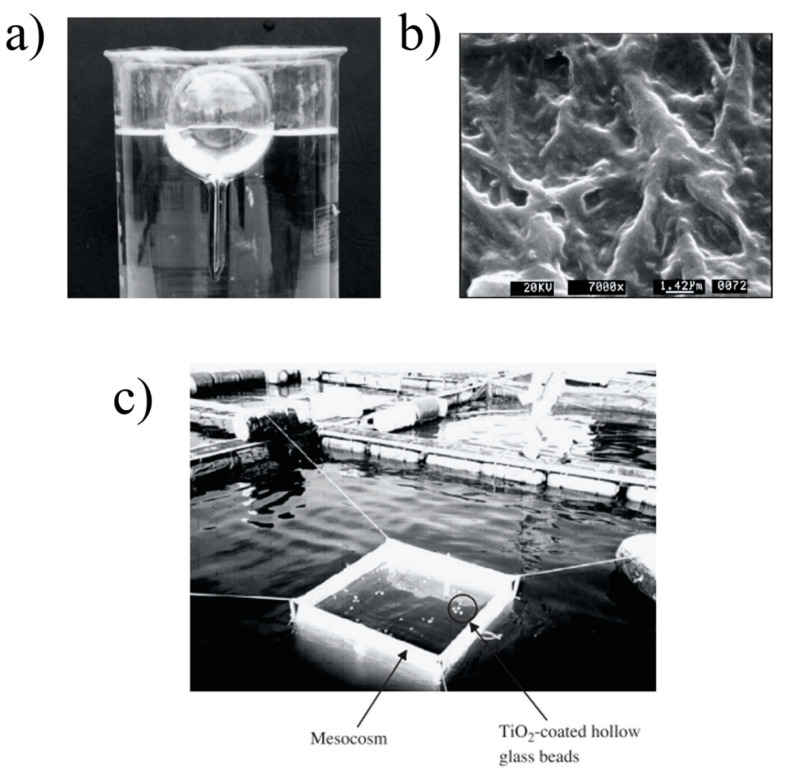
(**a**) Photo of a TiO_2_ hollow glass bead floating in water. (**b**) SEM images of a TiO_2_ hollow glass bead. (**c**) Photo of mesocosm at Nakdong River, Korea. Reprinted with permission from [155]. Copyright 2005, Elsevier.

Floating glass beads can also be used to prepare TiO_2_ floating structures. Algal problems in eutrophic water are serious and tend to result in the blocking of filters in drinking water supply facilities [155]. In addition, the presence of toxic cyanobacterial blooms in drinking water can lead to various human health problems [156]. Kim et al. (2005) prepared TiO_2_-coated hollow glass beads via a dip-coating method for the control of algal growth (Figure 10). The thickness of the TiO_2_ layer on the surface of the glass beads was 0.3 μm. Under the irradiation of UV-A light, *Anabaena* and *Microcystis* (cyanobacteria) lost their photosynthetic properties, while the string of *Anabaena* cells and the colonies of *Microcystis* cells were completely isolated into individual spherical ones. However, the TiO_2_-coated hollow glass beads displayed lower photocatalytic inactivation efficiency (60%) due to the presence of the inorganic siliceous wall surrounding the *Melosira* (diatom) cells. In a further real-world application, TiO_2_-coated hollow glass beads were inserted into a mesocosm installed at the Nakdong river (Kimhae City, Korea) (Figure 10). The results showed that vast amounts of chlorophyll-*a* were removed by the application of TiO_2_ glass beads [155].

According to Hosseini et al. (2007), even TiO_2_ immobilized on glass plates has excellent mechanical stability, with the leakage of TiO_2_ in their study being as low as 5 and 7% after the two reactions. In the same study, the authors immobilized TiO_2_ (Degussa P-25) on perlite granules for photocatalytic degradation of phenol. With a porosity of more than 95%, the granules easily floated on the water surface. The obtained XRD results showed that there were no significant changes to the TiO_2_ structure after the immobilization process. The uniform coating of TiO_2_ was confirmed by SEM images. In the photocatalytic reaction, 83.3% of 1 mM phenol was removed after 4 h under 125 W UV lamp irradiation. This was compared with 39.7% for the reaction under an 80 W UV lamp [38]. Hinojosa-Reyes et al. (2013) also used a similar coating method to coat indium-doped TiO_2_ (In-TiO_2_) on the surface of perlite granules for the gas-phase degradation of ethylbenzene in the plug flow reactor. The incorporation of In^3+^ into TiO_2_ frameworks prohibits the formation of hexacoordinated titanium and allows the creation of oxygen vacancies. A homogenous coating was achieved after 60 min. The coatings with 5% indium displayed enhanced photocatalytic activity compared to the undoped one [157].

In the study by Kim et al. (2005), TiO_2_ ceramic foam pellets were prepared using the dip-coating method. The three most significant steps were slurry preparation, foaming, and pelletizing. Silica powder (particle size: 3 μm) was used as the raw material in the fabrication of ceramic foam pellets. The pellets undergoing the final pelletizing process were sifted for constant size in the range of 3 to 5 nm and for close-to-spherical shape [158].

Aluminum (Al_2_O_3_) can be used as a supporting material due to its high surface area and abrasion resistance [53]. Mesoporous TiO_2_/γ-Al_2_O_3_ has been prepared using a combination of the sol–gel and oil-drop methods. The authors observed that the increase of TiO_2_ concentration led to the easily aggregated crystalline phase and rough surface formation on the composite granule (poor sphericity). The increase of TiO_2_ concentration also led to the formation of the rutile phase, in addition to the anatase phase. Additionally, the increase in the calcination temperature resulted in the increase of TiO_2_ crystallite sizes. In general, the optimal conditions for preparation of TiO_2_/γ-Al_2_O_3_ composite granules were: TiO_2_/(TiO_2_ + Al_2_O_3_) = 0.25, temperature calcination = 450 °C. The obtained composite granules had a diameter of around ~2 mm, the largest surface area measuring 306 m^2^/g and the smallest anatase crystalline size measuring 4.2 nm [53].

Han et al. (2009) prepared to float TiO_2_/polypropylene (PP) granules via hydrothermal methods at low temperature and to dope them with nitrogen by using trimethylamine (TEA) to activate photocatalysis under visible light. Acetic acid (AcOH) and acetylacetone (Acac) have been used as inhibiting agents in the preparation of TiO_2_ NPs. Between these two inhibiting agents, Acac can be used to dope nitrogen into a TiO_2_ lattice due to the stronger effects on the hydrolysis of the TiO_2_ precursor (titanium-n-butoxide), whereas AcOH cannot. Han et al. (2009) noted that the MO degradation by TEA-treated TiO_2_ with Acac under visible light was higher than by TEA-treated TiO_2_ with AcOH. However, when TEA-treated TiO_2_ with Acac was immobilized on PP granules, the photocatalysis was still lower than for the powder particles due to the low loading rate of TiO_2_ onto these granules, not to mention the small surface areas. Therefore, a strategy to improve loading rates should be found so as to improve the photocatalytic behavior of N-doped TiO_2_/PP granules [159]. In another study, Velasquez et al. (2012) coated TiO_2_ NPs (Degussa P25) on the surface of PP and low-density polyethylene (PE) at 153 °C and 106 °C, respectively, for 20 min. However, both PP-TiO_2_ and PE-TiO_2_ lost weigh due to the loss of TiO_2_ coating during the erosion test. The coated pellets showed high photocatalytic activity, with above 50% of 4-chlorophenol at the initial concentration of 100 ppm being removed after 6 h. The loading of PP-TiO_2_ and PE-TiO_2_ was 40 g. After 4 cycles, the photocatalytic activity of PP-TiO_2_ and PE-TiO_2_ was reduced to around 40% and 38%, respectively. The strong adsorption and accumulation of partially oxidized 4-chlorophenol intermediates on the active site of TiO_2_ could explain the results. They also suggested that the treatment under UV irradiation (220 nm) and H_2_O_2_ (10 wt%) could regenerate the TiO_2_ pellets [160].

Recently, Cunha et al. (2018) immobilized TiO_2_ on borosilicate glass spheres and applied them to a compound parabolic concentrator (CPC) for degradation of MB. The results showed that the detachment of TiO_2_ from the glass surface was very low (0.03%). The leaking of titanium into the water was analyzed by ICP-OES according to the standard method 3120. At 400 °C, the TiO_2_ was deposited onto the surface of the glass, without any changes in the characteristics of the photocatalyst. In the photocatalytic reaction, the TiO_2_ glass spheres removed 96% of the MB after 90 min and were recovered by washing in water under UV-vis irradiation. The TiO_2_ layers on the glass surface remained unchanged after five photocatalytic treatment cycles. The crystalline phase composition, crystalline size, BET surface area, and pore volume of TiO_2_, likewise, were nearly unchanged after the thermal treatment process [24].

In this section, various types of TiO_2_ macroscale structures have been introduced, including pure TiO_2_ pellets, composite TiO_2_ pellets, and immobilized TiO_2_ macroscale structures. In general, the preparation of TiO_2_ macroscale structures is more straightforward than TiO_2_ microscale structures. This makes TiO_2_ macroscale structures more comfortable to mass-produce. A number of methods have been used to prepare TiO_2_ macroscale structures, such as conventional compacting and pelletizing techniques, freeze-drying, and immobilization techniques. In addition, some advanced techniques and materials, such as HIPing and black TiO_2_, have also been utilized in the preparation of TiO_2_ macroscale structures.

## 4. Conclusions

TiO_2_ microscale structures and macrostructures have many advantages compared to TiO_2_ powders, such as their tunable structure, higher photocatalytic activity, and ease of recovery. TiO_2_ microscale structures are prepared from both TiO_2_ precursors (surfactants, hydrothermal or solvothermal techniques, or microwave techniques) and TiO_2_ NPs (spray-drying, freeze-drying, or immobilization techniques), while TiO_2_ macroscale structures are prepared mostly from TiO_2_ NPs (compacting and pelletizing techniques, freeze-drying, or immobilization). The advantages and disadvantages of TiO_2_ microscale and macroscale structures are briefly summarized below (Table 1).

For TiO_2_ microscale structures, solid spheres and hollow spheres share some similar synthesis methods, such as hydrothermal or solvothermal, surfactants, or templates. Recently, spray-drying, freeze-drying, pulsed laser ablation (PLAL), and microwave techniques have been used to prepare microscale structures. In some cases, solid and hollow TiO_2_ spheres could be obtained by adjusting the synthesis process [50,80]. In addition, the application of TiO_2_ microscale structures has been extended from wastewater treatment to other areas, such as controlled-release capsules, artificial cells, drug delivery, and even white-light-emitting diode (WLED) production [50,62]. However, due to the complexity of the synthesis process, microscale TiO_2_ spheres and hollow spheres are not easily mass produced. Therefore, simple and inexpensive methods of microscale TiO_2_ sphere preparation should be more thoroughly investigated.

In contrast, the preparation of TiO_2_ macroscale structures seems to be easier than TiO_2_ microscale structures. Different conventional methods, such as tumble growth, tableting, and extrusion, are still applied to prepare TiO_2_ pellets. The main limitation of these methods is the weak mechanical strength and low attrition resistance of the resultant pellets [53]. Recently, some studies have endeavored to improve the mechanical properties of TiO_2_ pellets by sintering them at very high temperatures (>880 °C) [52,123]. Additionally, freeze-drying and immobilization techniques for the preparation of pellets have been attempted. Immobilization of TiO_2_ on different substrates can lead to TiO_2_ macroscale structures with high mechanical stability. However, the leakage of TiO_2_ NPs into the surrounding environment is a critical problem. Recently, some advanced techniques, such as HIPing, along with advanced materials, such as black TiO_2_, have been used to prepare TiO_2_ pellets [1,32,54]. In the future, cheap and straightforward techniques, as well as new materials suitable for fabrication of macroscale TiO_2_ pellets high in both photocatalytic and mechanical strength, should be investigated more thoroughly. Additionally, the photocatalytic mechanism and properties of new materials such as the mentioned black TiO_2_ are still debated and should be clarified before any application to the preparation of TiO_2_ macroscale structures [161].

In conclusion, although TiO_2_ microscale and macroscale structures still face many problems, they still have considerable potential in a variety of areas due to their unique properties, especially their recycling efficiency. The recent efforts to overcome the limitations of these TiO_2_ structures should be continued and intensified.

## Figures and Tables

**Table 1:** Summary of TiO_2_ microscale and macroscale structures.

	Advantages	Disadvantages	Future Perspectives
TiO_2_ microscale structures	Solid TiO_2_ spheres and hollow TiO_2_ spheres could be obtained via modification of the synthesis process High surface area Tunable structure	Complex preparation methods	Inexpensive and straightforward methods should be found
TiO_2_ macroscale structures	Simple preparation methods Easy for mass production	Poor mechanical strength Low attrition resistance Immobilized TiO_2_ structures may have more mechanical stability, but the leakage of TiO_2_ to surroundings could be a critical problem	Simple, inexpensive methods to prepare TiO_2_ macroscale structures high in both photocatalytic and mechanical strength should be further investigated Novel materials should be applied to the fabrication of TiO_2_ macroscale structures
